# Keys to Expanding the Rural Healthcare Workforce in Kyrgyzstan

**DOI:** 10.3389/fpubh.2020.00447

**Published:** 2020-08-28

**Authors:** Paul Fonken, Inna Bolotskikh, Gulzhakhan Fazylovna Pirnazarova, Gulnura Sulaimanova, Shirin Talapbek kyzy, Aelita Toktogulova

**Affiliations:** ^1^Rural Health Project, Scientific Technology and Language Institute, Arlington, VA, United States; ^2^FM Department, Kyrgyz State Medical Institute for Retraining and Continuing Education, Bishkek, Kyrgyzstan; ^3^FM Department, Kyrgyz State Medical Academy, Bishkek, Kyrgyzstan

**Keywords:** rural training, rural education, professional support, rural retention, primary care, family medicine, rural generalist, former soviet union

## Abstract

**Objective/Background:** This study assessed Kyrgyzstan's progress with developing its rural primary care workforce and prioritized next steps to build on its current momentum. Kyrgyzstan has improved rural health care since 1997 through the implementation of family medicine, retraining of rural doctors and nurses, and other efforts. Attrition, emigration, urbanization, and population growth are threatening these hard-won advances. In response, Kyrgyzstan is now educating family medicine residents at rural sites and improving salaries. This study explores other steps to strengthen its rural health care, especially its rural generalists.

**Methods:** This was an observational study using a two-phase survey process. To access the current status of Kyrgyzstan's rural health care system, we surveyed key stakeholders within that system using a draft version of the new World Health Organization Rural Pathways Checklist. To prioritize next steps, we asked rural FM residents to rank the relative importance of 31 possible future actions to support Kyrgyzstan's rural primary care workers.

**Results:** Doctors and nurses involved in teaching rural health workers identified that Kyrgyzstan has made good progress with rural professional support and upskilling of existing health workers through education. They saw the least progress with selection of health workers and monitoring. The rural family medicine residents' top ten suggestions for rural recruitment and retention all involved improving working conditions (providing housing, internet, basic medical equipment, protected time off, better salaries, and more respect) and improving clinic efficiency (switching clinic scheduling from walk-in based to appointment based, optimizing the roles of clinical team members, and decreasing low-value clinic visits).

**Conclusions:** The WHO Rural Pathways Checklist helped to evaluate Kyrgyzstan's current efforts to promote rural primary care. The priorities listed above from the next generation of potential rural family doctors could help guide future steps to promote rural health in Kyrgyzstan and the Former Soviet Union.

## Introduction

### History of Family Medicine (FM) in Kyrgyzstan

After independence from the former Soviet Union in 1992, Kyrgyzstan adapted its health care system to meet some daunting challenges. At that time, they suffered a dramatic decline in funding and physician quantity, and they inherited a top-heavy health care system with relatively weak primary care. Like many other former Soviet States, Kyrgyzstan introduced FM to strengthen primary care (1). Many of the strengths of FM resulting from its evolution promised to address their specialty-driven fragmentation (2). Unfortunately, rural primary care outcomes research from this transitional time is sparse from these nations (3), especially from the Central Asian Republics. In 1997 The Kyrgyz State Medical Institute for Retraining and Continuing Education (KSMIRCE) introduced FM by establishing a FM training center in each of the seven oblasts (states) and trained a total of 63 FM trainers to staff these relatively rural centers. From 1999 until 2004 these KSMIRCE trainers retrained over 95% (2,691) of the country's outpatient physicians of various specialties to become “family group practice” doctors using a four-month curriculum (4, 5). During that same period, a similar parallel KSMIRCE program trained nurse trainers, who then retrained 85% (3,890) of the county's outpatient nurses to become FM nurses using a two-month curriculum. This retraining process is still active for doctors and nurses, and to date 6,212 nurses have been retrained as FM nurses (Pirnazarova G. Personal Correspondence). The KSMIRCE has also provide these nurses and doctors with many continuing education programs in every oblast over the years. Outside of the urban areas, these retrained doctors began practicing as generalists, caring for patients of all ages and both sexes with the help of the FM nurses. In the urban areas, however, these doctors did not significantly change their scope of practice, and health care delivery continued to be specialty driven. Initially, care significantly improved in the rural areas. In fact, in the 1990s, the infant mortality rate became lower in the rural areas than in the urban areas with a growing separation between the two rates at least through 2010 (6).

### Other Related Health System Projects

The Ministry of Health (MOH) is just starting its fourth national project to improve Kyrgyzstan's health care system: “Healthy Person—Prosperous Country (2019-2030) (7, 8). It emphasizes many primary care goals that will require a robust rural health care system. This emphasis on development of the regions outside of Bishkek is consistent with the National Development Strategy of the Kyrgyz Republic for 2018-2040 (9). Most of these national projects have had some input from international donors. The Swiss Development and Cooperation (SDC) has a long continuing history of projects to improve primary care in Kyrgyzstan (10). From 1997 through 2009, the United States Agency for International Development was very actively involved in broad longitudinal health system reform projects in Kyrgyzstan which included an emphasis on rural health (4).

### Rural FM Workforce Crisis in Kyrgyzstan

Unfortunately, the loss of health manpower through emigration and attrition and the lack of new rural family physicians have jeopardized the gains from these projects. Since 1998, Kyrgyzstan has trained over 500 new family doctors (4, 11) in the two main two-year residency programs based in the capital of Bishkek. Of these graduates, only ten ever practiced outside of the country's two major cities and only one is currently working rurally as family doctor (5). Rural primary care depends entirely on the retrained “family group practice” (FGP) physicians and over half of these doctors have emigrated or retired. The resulting rural physician shortage is complicated by a rapidly growing population. According to the head of the Association of Family Physicians and Family Nurses, Suyumjan Mukaeva, approximately half the population now lack reasonable access to a primary care doctor (12). In 2005 nationwide there was an average of 1,888 people per FGP doctor compared to 3,902 per FGP doctor in 2019 (4, 12). This ratio is uniformly worse in rural areas, reaching as high are 18,000 citizens per FGP doctor in some rayons (counties). To complicate this matter, in 2019 61% of the country's FGP doctors were beyond retirement age with 19% nearing retirement age (12). Fortunately, the supply of nurses is much better, even in rural areas.

### Ongoing Efforts to Promote Rural Primary Care

In response to this rural health manpower shortage, the MOH and other institutions are working hard to train more primary care workers and to make their work more attractive. From 2014 through the present, the Kyrgyz State Medical Academy (KSMA) has been reforming its curriculum to be more primary care oriented, with the help of the SDC (10). They admit medical students from many rural areas and have greatly expanded their annual number of FM residents from two in 2014 to 56 in 2018 (11). Since 2017, the MOH mandated that all medical school graduates complete 1-year of basic clinical training (general practice) in a rural hospital, which is included as part of the 2-year family medicine residency or the 3-year residencies for narrow specialties. Those interested in becoming FM specialists can continue to serve at that rural site for a second year. Currently, all 40 of the KSMIRCE FM residents and many from KSMA are training primarily in rural settings. These residents also function officially as part-time family doctors caring for attributed populations of 1,000–2,000 people. Their salary for this plus their monthly residency stipend totals <$80 per month. They may also work night shifts in the hospital or emergency departments for additional income. In contrast, residents in other specialties must pay a significant amount for their residency education. The first 24 FM residents who trained primarily in rural settings graduated in the summer of 2019. Unfortunately, many of these graduates have already been lost to follow up, and we were only able to locate three of these graduates who were still practicing in rural areas after their graduation.

### Study's Goals

This study gathered and analyzed opinions from rural FM graduates, current rural FM residents, and teachers of FM doctors and nurses to help policymakers more successfully recruit, prepare, and retain rural primary care workers.

## Methods

### Overview

This was an observational study using a two-phase survey process to first assess the current progress in developing a rural workforce and then identify priorities for further action.

#### Survey 1

To assess current progress, we surveyed a total of 71 national and regional level FM teachers (doctors and nurses) and rural clinical supervisors using the draft version of a new WHO self-assessment tool called the Rural Pathways Checklist (13) (https://www.globalfamilydoctor.com/site/DefaultSite/filesystem/documents/Groups/Rural%20Practice/19%20implementing%20rural%20pathways.pdf). Table 1 summarizes the selection process for our study's participants. This Rural Pathways Checklist self-assessment tool was developed by authors from Monash University in Australia in conjunction with the World Organization of Family Doctors (WONCA) at the request of the WHO, and it was ratified by the University of Queensland. We agreed to help the tool's authors to evaluate the usefulness of this new tool in a Russian-speaking area of Central Asia. The Rural Pathways Checklist consolidates evidence-based approaches for expanding rural health workforces in low and middle-income countries (14). It is based on the premise that successful placement and retention of rural healthcare workers depends on many different factors that should ideally be addressed in parallel. This tool uses a five-point Likert scale to evaluate progress with 30 steps along the pathway toward a robust rural healthcare workforce. These steps are organized into eight key domains, and the tool calculates a percentage grade for each domain, with 100% being full implementation of that domain within health care system. The draft tool includes five questions from the authors of the Rural Pathways Checklist to gather feedback about the draft checklist itself. We used a paper version in Russian to collect seven surveys, then converted this checklist to a Google Form in Russian, with the author's permission, sending it via “WhatsApp” (15, 16).

**Table 1:** Recipient selection for survey 1 (WHO rural pathways checklist).

**Institution**	**Kyrgyz State Med. Inst. For Retraining & Continuing Education**			**K. State. Med. Acad**.
	**Academic FM Physician Faculty Members**	**Academic FM Nursing Faculty Members**	**Rural FM Residency Clinical Supervisors**	**Rural FM Residency Clinical Supervisors**
# of survey recipients from this job category	20	12	7	32
Total # of professional in this job category	20	12	16	32
% of professionals surveyed in this category	100%	100%	44%	100%
Comments about the selection process	KSMIRCE is the only national institution tasked with continuing educaton for Kyrgyzstan's FM doctors and nurses		Convenience sample. KSMIRCE trains about 1/3 of the country's FM residents	KSMA trains about 2/3 of the country's FM residents

#### Survey 2

To prioritize potential next steps for recruiting and retaining the next generation of rural primary care workers, we created a list of 31 practical next steps using the eight main categories from the WHO Rural Pathways Checklist as a framework. We created another Google Form in Russian using a four-point Likert scale to assess the relative importance of these potential next steps: 0 = should not be done, 1 = low priority, 2 = medium priority, 3 = high priority (17). We then surveyed (via WhatsApp) all 24 of doctors who graduated in 2019 as the first class of rural FM residents and all 106 current rural FM residents. We ranked the potential future actions according to the average score from the participants and reviewed their free text comments to identify patterns.

## Results

### Response Rates

The overall response rate for survey 1 was 41% (29/71), with details summarized in Table 2. The response rate for survey 2 was 38% (40/106) for all current FM residents and 17% (4/24) for all rural FM residency graduates.

**Table 2:** Response rates for survey 1 (WHO rural pathways checklist).

**Institution**	**Kyrgyz State Med. Inst. for Retraining & Continuing Education**			**K. State. Med. Acad**.	
	**Academic FM physician faculty member**	**Academic FM nursing faculty member**	**Rural FM residency clinical supervisor**	**Rural FM residency clinical supervisor**	**Totals**
# of participants surveyed	20	12	7	32	71
# of fully completed responses	6	11	4	4	25
# of partially completed responses	1	0	3	0	4
Response rate for fully completed surveys	30%	92%	57%	13%	35%
Overall response rate (fully & partially completed surveys)	35%	92%	100%	13%	41%

### Evaluation of the Draft WHO Rural Pathways Checklist

62% (18/29) of those who did respond to survey one answered at least one of the five questions designed to evaluate the WHO checklist itself. Seventy-eight percent (14/18) thought the Checklist was applicable to their situation. Table 3 summarizes the evaluation results. Only 57% (4/7) people given the paper version of the survey successfully completed all the ranking questions, compared to all those who completed the electronic version. One person took the survey twice, 2 months apart with very consistent results, so we counted that as a single response, using the data from first survey.

**Table 3:** Opinions of 18 teachers who responded to the feedback questions about the WHO rural pathways checklist.

**Question**	**Summary of responses**
How well did the Checklist apply for your situation?	1 very applicable, 13 moderately applicable, 4 slightly applicable
What do you plan to work on now that you have assessed your rural pathway?	11 listed plans
Did it help to identify the gaps in your rural pathway?	9 stated yes
Did it help to identify the strengths in your rural pathway?	5 states yes
Do you have any feedback about the Checklist?	8 gave additional feedback (see below)
**Additional written feedback about the WHO rural pathways checklist**	
It is an interesting and useful survey	
Yes, It was difficult for me to understand the scoring system from one to five, which one means good?	
Yes, I did not understand the scoring system from one to five	
Some questions are too complicated	
Maybe the Checklist for nursing teachers should include other questions?	
It is sufficient	
Need to see in place	
It helped to identify almost all the weaknesses	

### Opinions of Rural Primary Care Teachers About the Current Situation

**Figure 1:**
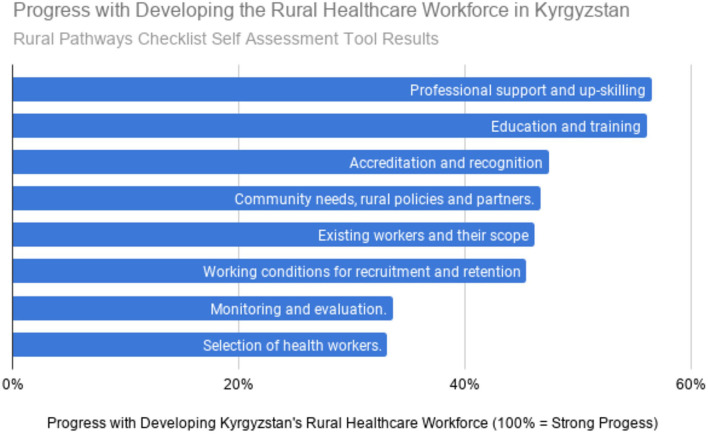
Progress with developing the rural healthcare workforce in Kyrgyzstan. Rural pathways checklist self assessment tool results.

About half of the FM teachers who responded live in rural areas and half in Bishkek, but all are involved with the education of rural doctors and nurses. Six of the doctors identified their position as either director or vice-director of their clinics. Table 2 summarizes response rates. Figure 1 documents the opinions of these teachers regarding Kyrgyzstan's progress with the eight main categories from the draft WHO Rural Pathways Checklist. They clearly felt the greatest progress has been in the areas of professional support, upskilling, education, and training. Table 4 summarizes their free text comments regarding the context, barriers, and enablers for each of the eight sections of the WHO Checklist. Thirty-four percent of these teachers (10/29) commented that salaries are too low and need to be improved to recruit and retain more rural health workers.

**Table 4:** Free-text comments about each section of the WHO checklist.

**WHO checklist categories ranked from most to least implemented**	**From doctors and nurses involved with teaching rural primary care**	**Number of similar comments**	
	**All respondents help with rural education: half live in the capital and half live rurally**	**Rural**	**Urban**
Professional support and up-skilling	Once we get direction from the MOH, we will begin upskilling nurses	2	
	Practitioners do not apply the newest clinical protocols and guidelines		1
	Theoretical knowledge is not always applied correctly in practice		1
	We are successfully training nurses nationally via internet about HIV care		1
Education and training	Lack of support for health ed.: infrastructure, supplies, safety, & steady work	3	1
	Local gov. & comm. are not always supportive of rural training. Trainees busy	1	2
	Our hosp./clinic uses an infant manikin, otoscope, AED& US to teach residents	3	
	Regional training centers provide good access to training		3
	We are doing distance ed and have plans for practical skills training		2
	Rural supervisors for residents: busy, poorly paid, lack experience as teachers		2
	Use more electronic distance education. Barriers: internet, computer literacy	1	1
	Rural residents get more clinical experience & may work after-hours for pay	1	
	Limit access to good clinical supervision & quality clinical medical references	1	
	The level of training during medical school is poor		1
	Rural FM supervisors are always available for the residents they supervise		1
	Regional training centers will need new teachers in the future	1	
Accreditation & recognition	Low prestige for family doctors. More prestige for narrow specialitsts	4	
	Graduates can practice their qualification in every medical facility	1	2
	Young doctors in rural areas need options for career growth & more training		1
	Not every facility can employ graduates or enable them to use all their skills		1
	Young doctors are often not recognized as professionals	1	
	Not all graduates are formally recognized by a qualification	1	
Community needs, rural policies and partners	Hosp./clinic is working with community regarding housing for residents	3	
	Government needs to set the plans for nursing, then we can implement them	1	2
	Health promotion & village health committees link clinics and the community	1	1
	There is little cooperation and communication with the rural communities	1	1
	Hosp/clinic is working with community council but it is not too effective	1	
	MOH policy needs to include 10x increase in rural worker salaries + benefits		1
	Urban teachers are limited in how often they can travel to teach rurally		1
	We have no external partners	1	
Existing workers and their scope:	Shortage of rural medical teachers & workers. They are too busy and quite old	2	3
	It is possible to attract young doctors by providing housing & med. Equipment	3	
	Important to continue to support retrained doctors since we have a shortage		1
Working conditions forrecruitment and retention	Low salaries for medical workers. This must be improved to retain them	6	4
	The work load is excessive for rural family doctors. Lack of protected time off	2	2
	Progress in these areas has been geographically spotty, affecting recruitment	2	1
	Lots of charting now (in electronic format)	1	
	Lack of medical equipment, tools for training and internet access		1
	Lack of free housing for residents is a barrier	1	
	Health professionals not safe within the system	1	
	Lack of kindergartens limits retension of young doctors in rural communities		1
Monitoring	Monitoring/supervision improves the quality of care. Helps young doctors	5	
	Barriers: lack of personel, time, money, training, equipment, & organization		3
	Monitoring does not lead to meaningful improvements		1
	Almost daily inspections from national, regional and district level agencies	1	
Selection of health workers	Important to choose active students from rural areas who want to return	3	1
	Rural residents training in their home towns now won't stay (low salaries)	1	

Rural FM residents' priorities for the future: Table 5 combines results from both surveys. The first column lists the eight main categories from the Checklist as ranked by the teachers, with the most fully implemented categories at the top and least implemented categories at the bottom. The second column of lists the potential specific actions that Kyrgyzstan could take in the future to strengthen rural health care. The table uses three colors to reflect the relative importance of each action according to the rural FM residents and graduates. These same actions are ranked in Table 6 according to the average score assigned to each by the residents and graduates. Table 7 lists the resident's free-text comments. Resident's free-text comments mirrored their numerical opinions with eleven commenting on poor financial support and five residents expressing frustration over the lack of respect from patients. Some noted that patients seem to have more rights than doctors.

**Table 5:** Potential specific future actions to improve the kyrgyzstan's rural health workforce.

**WHO checklist categories ranked by teachers from most to least implemented**	**Ranked by rural family medicine residents by color**		
	**Yellow = High Priority (10 highest ranking future actions per rural FM residents)**	**Green = Medium Priority**	**Brown = Low Priority (10 lowest ranking future actions according to rural FM residents)**
Professional support and up-skilling	Incentivize ongoing training		
	Reward all care team members for improving their patient populations health outcomes		
	Provide regular tele-conferencing opportunities with peers regionally and/or nationally		
	Provide telemedicine support from key specialists		
	Improve access to evidence-based medical references in Russian		
Education and training	Improve training of medical students in primary care skills		
	Continue to train FM residents at rural sites		
	Improve the clinical training of FM residents so they are better prepared for their roles		
	Improve the clinical training of nurses to prepare them for their expanding roles		
	Create an on-site salaried program to train rural FM residency graduates as teachers		
Accreditation & recognition	Recognize and honor rural health workers for their valuable role in the health care system		
Community needs, rural policies, and partners	Train rural health workers and administrators more about existing rural healthcare policies		
	Increase the involvement of rural health workers and administrators in national policymaking		
	Shift national and regional governmental policies to be more favorable for the rural health care system		
	Increase community engagement with their health and the health care system		
Existing workers and their scope:	Expand the roles for family medicine nurses and feldchers		
	Better define the roles for rural FM doctors, specialists, nurses and pharmacists		
	Strengthen the roles of social workers		
	Strengthen the roles of village health committees		
Working conditions for recruitment and retention	Improve salaries for residents, family medicine doctors and nurses in rural areas		
	Extend the rural doctor's deposit program, which currently rewards after 3 years of service		
	Provide quality housing and internet access for rural FM residents and doctors		
	Decrease the charting and reporting burden for doctors		
	Change policies that result in low-value clinic visits		
	Provide adequate time off		
	Protect doctors from afterhours responsibilities		
	Create an appointment system for clinic visits		
	Provide adequate basic equipment in every clinic		
Monitoring	Shift monitoring from an intrusive punitive process to an efficient supportive process		
Selection of health workers	Continue to admit significant numbers of nursing and medical students from rural areas		
	Strengthen rural secondary school education to better prepare students for medical careers		

**Table 6:** Potential specific future actions to improve the kyrgyzstan's rural health workforce.

**Average Rating on 0-3 Scale**	**Ranked by Rural Family Medicine Residents and Recent Graduates**
2.62	Provide quality housing and internet access for rural FM residents and doctors
2.62	Provide adequate basic equipment in every clinic
2.61	Improve salaries for residents, family medicine doctors and nurses in rural areas
2.59	Protect doctors from after-hours responsibilities
2.57	Create an appointment system for clinic visits
2.50	Better define the roles for rural FM doctors, specialists, nurses and pharmacists and how they can work together
2.46	Recognize and honor rural health workers for their valuable role in the health care system
2.41	Change policies that result in low-value clinic visits
2.41	Provide adequate time off
2.38	Improve access to evidence-based medical references in Russian
2.27	Strengthen rural secondary school education to better prepare students for medical training programs
2.27	Provide regular tele-conferencing opportunities with peers regionally and/or nationally
2.24	Create an on-site salaried program to train rural FM residency graduates as teachers
2.24	Reward all care team members for improvements in the health outcomes of their patient population
2.24	Provide telemedicine support from key specialists
2.24	Incentivize ongoing training
2.22	Extend the rural doctor's deposit program, which currently rewards them after 3 years of service
2.19	Shift monitoring from an intrusive punitive process to an efficient supportive process
2.16	Improve the clinical training of FM residents so they are better prepared for their roles
2.15	Expand the roles for family medicine nurses and feldchers
2.14	Improve the clinical training of nurses to prepare them for their expanding roles
2.13	Strengthen the roles of social workers
2.08	Strengthen the roles of village health committees
2.08	Improve training of medical students in primary care skills
2.00	Decrease the charting and reporting burden for doctors
1.95	Increase community engagement with their health and the health care system
1.92	Continue to admit significant numbers of nursing and medical students from rural areas
1.89	Increase the knowledge of existing rural healthcare policies among rural health workers and administrators
1.84	Shift national and regional governmental policies to be more favorable for the rural health care system
1.68	Increase the involvement of rural health workers and administrators in national policymaking
1.55	Continue to train FM residents at rural sites

**Table 7:** Rural family medicine resident's free-text comments.

**WHO checklist categories**		**Number of similar comments**
Education and training	Virtual professional networking would helpful	3
	Virtual professional networking would not be helpful or practical	3
	Need more easy accessible medical literature in family medicine	2
	Need for more respect	1
	There are few well-educated managers	1
	Improve the quality of education in nursing schools	1
	Qualified teachers and doctors do not stay in the countryside	1
	Need continuous medical education	1
	Resident motivation would improve with better salaries	1
Community needs, rural policies and partners	Barriers: Corruption among government officials	1
	Rural communities are not motivated to cooperate	1
	Need to inform the population about the work of the family doctor	1
	Low awareness of officials	1
	Villagers are poorly educated	1
	Rural health care is very important	1
	It is important to educate the population about chronic diseases	1
Working conditions for recruitment and retention	Lack of financial support, poor salary	11
	Lack of respect for doctors and their rights and opinions	4
	Ungrateful and demanding population	3
	Poor working conditions	3
	Poor facilities and equipment	2
	Poor internet access	2
	High workload	2
	Money is not the only tool to retain our young specialists	1
	Unsafe working environment	1
	The government should provide affordable housing for medical workers	1
	Lack of quality education for children of rural medical workers	1
	Important to provide a good living and training conditions for residents	1
Monitoring	Monitoring is important and improves rural health care	3
	Monitoring does not improve rural health care	2
Selection of health workers	Encourage rural secondary school students to pursure medical careers	1
	Provide more scholarships to rural students for medical education	1
	Be of use in the country where you were born	1
	Entrance requirements to medical schools must be equal for everyone	1

## Discussion

### Professional Support and Upskilling Rural Health Workers

Not surprisingly the FM teachers consider professional support and upskilling of existing rural health workers as the most fully implemented aspect of the country's plan to support rural health, since they have accomplished a lot in this area. However, the benefits of the retraining and continuing education programs for FGP doctors and nurses are rapidly eroding as these rural FGP doctors and nurses retire and/or emigrate. The FM residents identified the importance of creating a rural faculty development program and providing regular educational opportunities, including educational teleconferencing for peers. These and many additional actions as listed below will be required to successfully develop the next generation of rural health workers and their teachers.

### The Power of Urbanization

The residents surveyed ranked rural residency training as the least important of the 31 future next steps to increase the number of rural health workers. Likely, this reflects the strength of the country's tendency toward urbanization. Whereas, the standard of living in the capital (Bishkek) has changed dramatically since independence, it has changed relatively little in the rural areas. The rural economy is very weak, and ~20% of the country's citizens are currently working outside the country. One of the residents commented, “It's impossible to retain what is flowing. It's not just the doctors who are leaking, but the population, youth, entire families. This question is not for me, but for the politicians.” The strength of these demographic trends calls for dramatic interventions to promote the rural healthcare workforce in Kyrgyzstan. Hopefully, the opinions capture in the study will help guide these interventions.

### The Importance of Improving Working Conditions

The rural FM residents ranked improving working conditions as the most important next step in potentially recruiting them to serve in rural areas after graduation. They placed the highest priority on housing with Internet access and better equipment in the hospitals and clinics. As of 2019, none of the 30 rural FM residents visited by Dr. Fonken around the country had their own otoscope, and most did not have easy access to an otoscope. Most of the sites did not provide housing for residents. The residents' next highest priority was improving the salary. Although monthly salaries for family doctors have risen from about $20 in 1997 to $200–400 in 2019, they are still inadequate to provide a reasonable lifestyle. The government is also providing a monthly bonus (about $14) to doctors who work above a certain altitude (80% of the country is mountainous) and a quarterly bonus (about $400) for young doctors who remain in the most underserved areas for up to 3 years (18). Respondents ranked expanding this bonus program as seventeenth in importance out of 31. The residents also highly valued some relatively inexpensive solutions: decreasing the charting/reporting burden, providing protected time off, decreasing the number of low-value visits, changing monitoring from an intrusive punitive process to a more efficient supportive process, and implementing an appointment system. The MOH is currently implementing an electronic appointment system (19), but most patients still prefer to walk in, even when an appointment system is available. This is one of many areas that will require behavioral change on the part of the patient population, to improve the lives of rural health workers. Overall, the residents' priorities are in line with the goals of the current MOH-SDC project on non-communicable diseases, which stated that the primary care system in Kyrgyzstan must be able to “offer better salaries and career prospects to medical personnel to reduce migration to Russia and Kazakhstan and to provide incentives for family doctors to settle in rural areas”: (20).

### Task-Shifting in Primary Care

These residents also desire better defining the roles of various types of rural health care workers (seventh most important step). The independent Association of Family Physicians and Family Nurses has been working hard with the MOH to do this. The residents were more in favor of expanding the role of nurses rather than shifting tasks to social workers and village health committees. This may stem from the fact that the residents work more closely with the nurses, and do not interact much with social workers, public health nurses, or village health committees, despite them being well-established as part of the rural health care system. In rural Kyrgyzstan, nurses are providing an increasing proportion of the care as the number of physicians continue to decrease. Almost half of the respondents to our WHO survey were nursing teachers from around the country. They felt that there has been less overall progress with promoting rural primary care than their physician colleagues. The nurses ranked average overall progress along the Rural Health Pathway as only 36%, compared to a 57% ranking from the doctors. The nursing teachers' comments confirmed many of the same problems, needs, and priorities as the physicians. Several of the nursing teachers expressed that they are anxiously awaiting direction from the MOH. Fortunately, the MOH is about to finalize a plan to expand nursing education and roles. The nurses were also appreciative of an ongoing ICAP/PEPFAR project to train rural nurses about HIV care (21) and efforts by the SDC/MOH to revise the national curricula for nursing training (10). The need for shifting tasks from doctors to nurses and improving working conditions in rural primary care was confirmed by a recent WHO report. It listed the following two policy considerations among their eleven suggestions for improving health services delivery in Kyrgyzstan: (1) “Revisiting the capacity of FM doctors and nurses” and (2) “Improving the attractiveness of FM practice through financial and institutional incentives” (22).

### Coordinating With Communities and Other Partners

FM teachers expressed that Kyrgyzstan's health care system should improve coordination with rural communities and other partners. Kyrgyzstan has benefited from many partnerships to strengthen rural primary care. The interface between large projects and community-based initiatives has accomplished some of the tasks that are most valued by rural FM residents, such as supplying free housing and internet. The hospital and clinic in the small town of Kyzyl-suu (Issyk-kul oblast) used a community grant to remodel a building into a dormitory for rural FM residents. They also linked to a project involving the SDC and MOH to better equip the hospital and clinic, including adding medical equipment, upgrading their internet and implementing an electronic medical record (10). In the fall of 2019, Dr. Fonken spent 5 weeks there exchanging professional experience with their four rural FM residents and their local clinical supervisors and administrators. Such professional exchanges are a central part of a longstanding cooperative arrangement between the KSMA, the KSMIRCE and Scientific Technology and Language Institute (STLI), a non-governmental humanitarian organization. Dr. Fonken found the medical team in Kyzyl-suu working effectively with enthusiasm. The residents seemed pleased to have free housing and internet access. Their morale was good, and they liked having ample clinical experience and responsibility. There are similar examples of health facilities providing housing to residents in the Narin and Batken oblasts through creative partnerships with the community and other partners. These are good examples for other communities to follow.

### Selection of Health Workers

The WHO Rural Pathways survey participants identified the selection of health workers as a current area of weakness. Surprisingly, the residents ranked this as a low priority for future efforts, even though many of the current residents are from the rural area they are now serving. Certainly, in the US literature, recruiting medical students with a rural background is a key predictor of future rural medical practice (23). In personal interviews Dr. Fonken found that many rural residents with children were pleased to be serving their small home communities, where they had family available to help with housing and childcare. The residents in our survey felt that improving secondary education in the rural areas is a moderate priority, and some expressed that the quality of rural schools and lack of kindergartens is a barrier to choosing a career in rural FM.

### Suggestions Regarding the Draft WHO Rural Pathways Checklist

We were able to use this new WHO tool to identify Kyrgyzstan's relative progress with various steps along a pathway toward a more robust rural healthcare system. The Rural Pathways Checklist seemed to help participants to think broadly about their situation in Kyrgyzstan. Most of the respondents found the tool applicable and many found it helpful in identifying gaps in their approach to strengthening rural health care. The Google Forms version was more effective than the paper version. Almost half of the participants who used the paper version of the checklist failed to complete all the ranking questions, and many of them expressed confusion about whether the tool pertained only to that person or to the healthcare system more broadly. We clarify this in the electronic version by adding the phrase “how Kyrgyzstan's health care system is performing in these areas.” This tool is likely to be useful in other similar countries. However, if Russian is a second language for recipients, the translation should be simplified. We used the free-test questions from the WHO tool in survey two, however, several of the residents complained that the Russian language used was hard to understand. Two of the Kyrgyz authors also expressed a concern that the translation was in “high academic Russian.” Finally, we recommend simplifying the scoring method by dropping the use of percentages and by switching to a zero to three scale (no progress, weak progress, moderate progress, and strong progress), since it fits better on a phone screen.

### Limitations

Conclusions from this observational study are limited due to the demographics of the sample groups and to the relatively low response rate. Although we did survey most of Kyrgyzstan's teachers of family medicine doctors and nurses, we failed to include any high-level health system policy makers, who would likely have expressed a different perspective. The inconsistency of telecommunications in rural Kyrgyzstan and linguistic challenges probably adversely influenced the response rate. The response rates do not allow us to make any statistically significant conclusions, however, the observations collected are still valuable. The residents' priorities regarding recruitment and retention are generally in-line with other similar studies (23). Our hope is that these qualitative observations will lead to more rigorous quantitative studies regarding rural workforce issues in the Former Soviet Union.

## Conclusion

The WHO's Rural Pathways Checklist helped primary care teachers in Kyrgyzstan to evaluate how Kyrgyzstan is supporting its rural health care system. They have progressed the most in the areas of professional support and upskilling/education of rural health workers. They have made the least progress with working conditions, monitor, and section of rural health workers. Current and recent rural FM residents emphasized the importance of improving working conditions (providing housing, Internet, basic medical equipment, protected time off, better salaries, and more respect) and improving clinic efficiency (switching clinic scheduling from walk-in-based to appointment-based, optimizing the roles of clinical team members and decreasing low-value clinic visits). These observations can help guide policymakers' responses to the current rural health manpower crisis faced by Kyrgyzstan and neighboring countries.

## Data Availability Statement

The original contributions presented in the study are included in the article. Inquiries can be directed to the corresponding author. The raw data supporting the conclusions of this article will be made available by the corresponding author, without undue reservation, either in English or Russian.

## Ethics Statement

Ethical review and approval was not required for the study on human participants in accordance with the local legislation and institutional requirements and written (electronic) informed consent to participate in this study was provided by the participants.

## Author Contributions

PF design of study and surveys, initial data analysis, and writing initial manuscript draft. IB, GP, GS, ST, and AT selection of survey participants, wording, distribution of surveys, and final review of data and manuscript. All authors contributed to the article and approved the submitted version.

## Conflict of Interest

The authors declare that the research was conducted in the absence of any commercial or financial relationships that could be construed as a potential conflict of interest.

## Abbreviations

FM: family medicine
ICAP: International Center for AIDS Programs—Columbia University
KSMA: Kyrgyz State Medical Academy
KSMIRCE: Kyrgyz State Medical Institute for Retraining and Continuing Education
MOH: Ministry of Health of the Kyrgyz Republic
PEPFAR: President's Emergency Plan for AIDS Relief
SDC: Switzerland Agency for Development and Cooperation
STLI: Scientific Technology and Language Institute
WHO: World Health Organization.
